# Emotions in Everyday Life

**DOI:** 10.1371/journal.pone.0145450

**Published:** 2015-12-23

**Authors:** Debra Trampe, Jordi Quoidbach, Maxime Taquet

**Affiliations:** 1 Department of Marketing, Faculty of Economics and Business, University of Groningen, Groningen, The Netherlands; 2 Department of Economics and Business, Pompeu Fabra University, Barcelona, Spain; 3 Computational Radiology Laboratory, Boston Children’s Hospital, Harvard Medical School, Boston, United States of America; University of Bologna, ITALY

## Abstract

Despite decades of research establishing the causes and consequences of emotions in the laboratory, we know surprisingly little about emotions in everyday life. We developed a smartphone application that monitored real-time emotions of an exceptionally large (N = 11,000+) and heterogeneous participants sample. People’s everyday life seems profoundly emotional: participants experienced at least one emotion 90% of the time. The most frequent emotion was joy, followed by love and anxiety. People experienced positive emotions 2.5 times more often than negative emotions, but also experienced positive and negative emotions simultaneously relatively frequently. We also characterized the interconnections between people’s emotions using network analysis. This novel approach to emotion research suggests that specific emotions can fall into the following categories 1) *connector emotions* (e.g., joy), which stimulate same valence emotions while inhibiting opposite valence emotions, 2) *provincial emotions* (e.g., gratitude), which stimulate same valence emotions only, or 3) *distal emotion*s (e.g., embarrassment), which have little interaction with other emotions and are typically experienced in isolation. Providing both basic foundations and novel tools to the study of emotions in everyday life, these findings demonstrate that emotions are ubiquitous to life and can exist together and distinctly, which has important implications for both emotional interventions and theory.

## Introduction

Hundreds of papers in psychology, medicine, marketing, management, and many other fields begin by asserting that emotions are ubiquitous to human life. But exactly how “ubiquitous” are they? A tremendous body of work has established that various stimuli and situations can cause emotions [1–4] and that once people experience emotions, it guides their thoughts and behaviors [5, 6]. However, despite decades of research establishing the causes and consequences of emotions in the laboratory, we know surprisingly little about emotions in real life. That is, how many hours a day do we feel happy, in love, fearful, or disgusted? What specific emotional state should we seek to offset a burst of anger? Is gratitude really an antidote for sadness? Answering these fundamental questions about the frequency and centrality (i.e., interconnectedness) of emotions in everyday life is crucial to our understanding of human experience and may guide research and interventions in important ways. In the current research, we report the first “big data” account of how people actually experience emotions in real-time in their everyday life. Bringing together network science and emotion research for the first time, we use network analysis to elucidate interrelations between emotions. This approach provides new insights into our everyday emotional life.

Recent years have witnessed an explosion of research on specific emotions. In particular, a fast growing body of work aims to investigate the health benefits of specific emotions such as gratitude [7], awe [8], and love [9] and psychological interventions that encourage people to cultivate these specific emotions are currently expanding [10, 11]. Examining the effect of specific emotions is also a very hot topic in behavioral economics, and researchers have started to uncover how different emotional states influence judgment and decision-making. For instance, the experience of joy and anger tends to boost people’s tendencies to take actions, fear exacerbates perceptions of risk, and disgust can increase people’s desire to discard their belongings, even when the source of these emotions is unrelated to the situation at hand [12]. These exciting advances in our understanding of different specific emotions contrast with how little we know about how these emotions are experienced in everyday life. Only a handful of studies have attempted to track people’s emotions in natural settings and they have typically done so by providing small samples (from a couple dozens to a couple hundreds) of undergraduates or local community members with pagers, which prompted participants to record whenever possible their feelings on a paper diary during random points in the day [13–16]. These initial studies provided somewhat disparate findings. Some researchers report that happiness and relaxation are the most frequent human emotions [16], whereas others find that anxiety and excitement dominate our emotional life [14]. These incongruent results may not be surprising, however, given the very small sizes and idiosyncratic characteristics of the samples that have been used in the past. Moreover, the use of retrospective measures makes it difficult to ensure that participants report on their emotions at the moment they are being experienced, thereby potentially introducing memory-related biases. What is currently needed is a large-scale investigation of human emotions in a large and diverse sample of people using a precise measurement tool that allows for more reliable and generalized conclusions about their everyday experience of emotions. Accordingly, we drew from the literature on Experience Sampling Methods (ESM) and Ecological Momentary Assessment (EMA) [17] to construct a tool that makes it possible to record people’s emotions as they go about their daily lives.

Beyond frequency, the wide range of emotions people can experience prompts the question of how the main specific emotions are interrelated. For example, is anger more likely to be experienced in tandem with anxiety or with sadness? Can we feel love and contempt at the same time? We know very little about which emotions typically co-occur or are rarely or never experienced in tandem. Existing research on the structure of affect has primarily provided insight into the factors that may underlie emotions. For instance, the best-known model of affect is the circumplex model, which proposes that emotions can be ordered on the circumference of a circle that comprises two orthogonal psychological dimensions: valence and arousal—the distance between two specific emotions corresponds to the similarity and correlations between them [18–20]. For example, according to the circumplex model, the emotion “fear” falls in the high arousal/negative valence quadrant of the circle, while “satisfaction” falls in the low arousal/positive valence quadrant. Multidimensional scaling of similarity judgments of emotions has provided support for the proposition that valence and arousal serve as the primary dimensions of emotions [21]. However, using multidimensional scaling and factor analysis to examine the interrelations between emotions essentially simplifies the space of emotions by attempting to elucidate common factors that underpin their variability. In the present investigation, we aim to enrich research on emotion by taking a novel complementary approach to studying the relationships between different specific emotions from a network perspective. Our approach based on the theory of complex networks fully encodes the complexity of everyday emotional life. Factor analysis, an approach that has been used in earlier studies, makes the fundamental assumption that emotional space can be reduced to smaller number of dimensions. In a seminal paper [22], the authors introduce network analysis for sociometric data because “Clearly, the standard tools of regression, discriminant, or factor analysis are not readily applicable.” (p. 512). Graph analysis does not make the assumptions that factor analysis makes. It simply represents the complexity of interactions between different elements of a network. In the current study, we show that network analysis provides new insights into the centrality of specific emotions and their relation with other emotions.

Several methodological challenges have made studying the frequency and centrality of emotions as they are experienced in everyday life with a large and diverse group of people a particularly difficult endeavour. We sought to overcome these challenges by developing a multiplatform experience sampling smartphone application, which yielded real-time measures among an exceptionally large group of people. This approach allowed us to examine three fundamental questions about human emotions: 1) how often do people experience emotions in general, 2) which emotions do people specifically experience (i.e., frequency), and 3) how central are different emotions within the emotion network (i.e., centrality)?

## Method

### Study design and participants

Participants volunteered for the study by downloading “58 seconds”, a free francophone mobile application for both iPhone and Android dedicated to measuring various aspects of users’ psychological experience through short questionnaires presented at random times throughout the day. The project received significant coverage on national television in both France and Belgium, totalizing over 60,000 users and half a million questionnaires completed.

We developed an emotion questionnaire that was embedded in a larger study. Specifically, participants were asked to indicate whether or not they were currently feeling nine specific positive (alertness, amusement, awe, gratitude, hope, joy, love, pride, and satisfaction) and nine specific negative (anger, anxiety, contempt, disgust, embarrassment, fear, guilt, offense, and sadness) emotional states, which were adapted from the modified Differential Emotion Scale (mDES) (mDES: [23]) and its French translation [24]. Participants also had the opportunity to indicate that they were not currently experiencing any emotion. Specifically, the question in the emotion list read: *“Are you currently feeling one or several of the following emotions*. *If you are not currently feeling any of these emotions*, *simply click ‘Next’*.*”* Although there has been extensive debate in literature about which emotions should be considered fundamental and which emotion might be more secondary, and whether there is such thing as a fundamental emotion [25], our choice of emotion terms was guided by three considerations. First, these 18 emotions come from a validated scale [26,27] and the scale is one of the most widely used scales of discrete emotions [28,29]. Second, each of these 18 emotions has been extensively studied (ranging from 1,790 articles with “embarrassment” in the title to 1920,000 articles with “satisfaction” in the title according to a Google Scholar search in May 2015).

**Fig 1:**
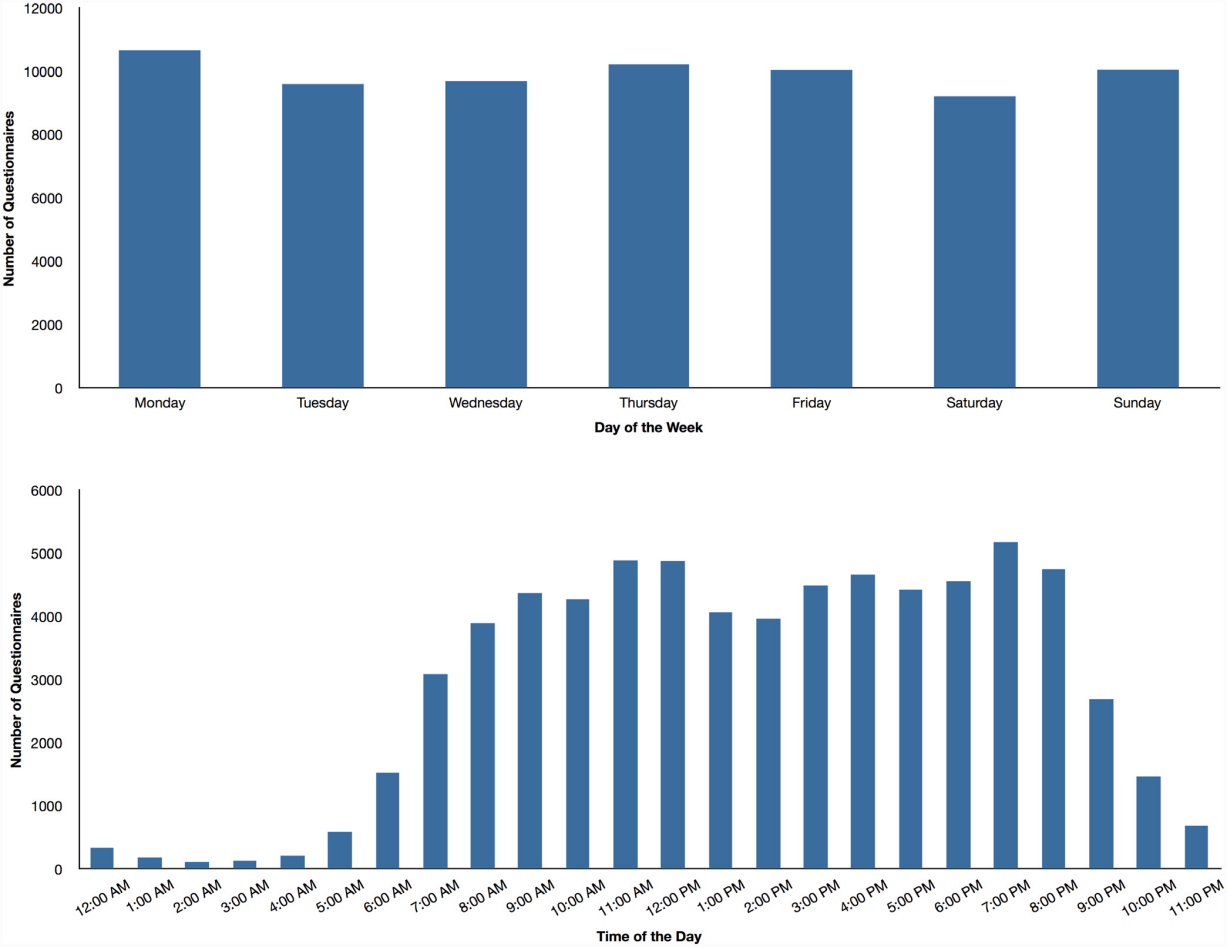
Completed questionnaires. Number of questionnaires completed during different days of the week and times of the day.

The present sample represents over a year of data collection (February 2013 to April 2014). Note that the application is still running and more data are currently being collected. In total, 11,572 participants (M_age_ = 32.9, SD_age_ = 10.4; 75% women) completed a total number of 65,721 emotion reports over an average of 35 days (median = 8.6). Table 1 and Fig 1 display more information about the sample.

**Table 1:** Sample characteristics.

**Variable**	**Descriptives**
**Age**	*M* = 32.9; *SD* = 10.43; range = 14–74 years
**Gender**	75% female (N = 49,039), 25% male N = 16,682)
**Nationality**	93% French; %% Swiss; 2% other; 0,5% Belgian
**Number of responses**	*M* = 5.7; *SD* = 9.6; range = 1–257 times
**Time of day of response**	*M* = 3:26 PM; SD = 4.45; mode = 9:19 AM, range = 00:00–24:00

Participants were given no financial compensation but were provided once a week with feedback on their aggregated levels of emotions. Participants answered several demographic questions, and were asked which days of the week and within what time windows they wished to receive questionnaire requests (default = 7 days/week from 9 AM to 10 PM). Participants could also customize the number of daily questionnaire requests they wished to receive (default = 4, minimum = 1, maximum = 12). The application algorithm then chose random times to send questionnaires within each participant’s day, with a minimum of one hour between two questionnaire requests. The random sampling was ensured through a notification system that did not require users to be connected to the Internet. New random times were generated each day, and the times were independently randomized for each participant. Thus, although participants were free to choose the time window in which they received questionnaires, the exact times they received a questionnaire was randomly determined, with at least one hour between two questionnaires. At each of these times, participants received a notification on their mobile phone informing them that a new questionnaire was available. They then had the possibility to take the questionnaire, snooze it (i.e., delay the questionnaire request by 9 minutes), or reject it.

We attempted to avoid some of the pitfalls of potential self-selection bias posed by smartphone research, including a potentially younger and wealthier sample than the average population, by advertising our research project on various channels—from local newspapers to national primetime television—and by making our experience sampling application a multiplatform one. That is, while other applications are exclusively developed for the relatively expensive iPhone (see e.g., www.mappiness.org.uk and www.trackyourhappiness.org), our application was available for all smartphone types. In addition, the application was designed such that it could function without frequent access to Internet; data kept being time-stamped and directly stored on the participants’ phone until being uploaded to a secure server whenever an Internet connection was available. This unique off-line function further increases the ecological validity of emotion reports because participants could virtually answer emotion questions anytime and everywhere. Taken together, these features allowed us to potentially reach over 40% of the French population [30].

The Ethics Committee of the University of Groningen, the Netherlands, approved the study in written form. The study method was carried out in accordance with the approved guidelines. All study protocols were approved by the aforementioned Committee. At initial signup, participants provided their written informed consent. The data have been deposited on Open Science Framework (https://osf.io/h2jp3/?view_only=c685ecbec4f54afeb8046af8980afd4c).

## Results

### Frequency of Emotions

#### Emotions in general

To take into account the nested structure of our data (most participants answered multiple times), estimations of the frequency of emotions were obtained using multi-level modelling with a compound symmetry covariance matrix. We computed the percentage of the time that people reported feeling an emotion; that is, the frequency of moments for which participants indicated they experienced at least one of the 18 emotions on the list. On average, people reported experiencing one or several emotions 90% of the time. Specifically, participants indicated experiencing one or several positive emotions (i.e., positive emotions only) 41% of the time, one or several negative emotions (i.e., negative emotions only) 16% of the time, and at least one positive and one negative emotion simultaneously (i.e., mixed emotions) 33% of the time. Breaking down these results by emotion, we computed the frequency of moments for which participants indicated they experienced each of the 18 distinct emotions on the list. As depicted in Table 2, the top 3 most frequently experienced positive emotion was *joy* (35% of the time), followed by *love* (30% of the time), and *satisfaction* (27% of the time). In terms of negative emotions, the most frequently experienced emotion was *anxiety* (29% of the time), *sadness* (20% of the time, and *disgust* (11% of the time). In terms of mixed emotions, the emotions that most frequently co-occurred with an opposite valence emotion were anxiety and love. For further details about mixed emotions, please refer to S1 Table.

**Table 2:** Frequency and centrality of everyday emotional experience.

	Frequency			Centrality
Emotion	Percentage	95% CI Lower Bound	95% CI Upper Bound	Degree Centrality
Joy	35	34	35,2	3
Love	30	29,3	30,6	1,4
Anxiety	29	28,6	29,8	1,7
Satisfaction	27	26,5	27,6	2,7
Alertness	24	24	25	1,3
Hope	22	21,8	22,9	2
Sadness	20	19,7	20,8	2,6
Amusement	16	15,9	16,8	1,8
Pride	13	12,7	13,6	2,2
Disgust	11	11	11,8	2,3
Anger	10	9,5	10,2	2,2
Gratitude	9	8,6	9,4	2,1
Guilt	5	5,2	5,7	1,6
Fear	5	5,1	5,7	1,6
Awe	5	4,9	5,5	1,9
Offense	5	4,7	5,2	1,8
Embarrassment	5	4,4	4,8	1,2
Contempt	1	1	1,2	1
Positive emotion only	41	40,1	41,4	
Negative emotion only	16	15,7	16,6	
Mixed emotion	33	32,3	33,6	
ANY EMOTION	90%	89,3	90,41	

**Fig 2:**
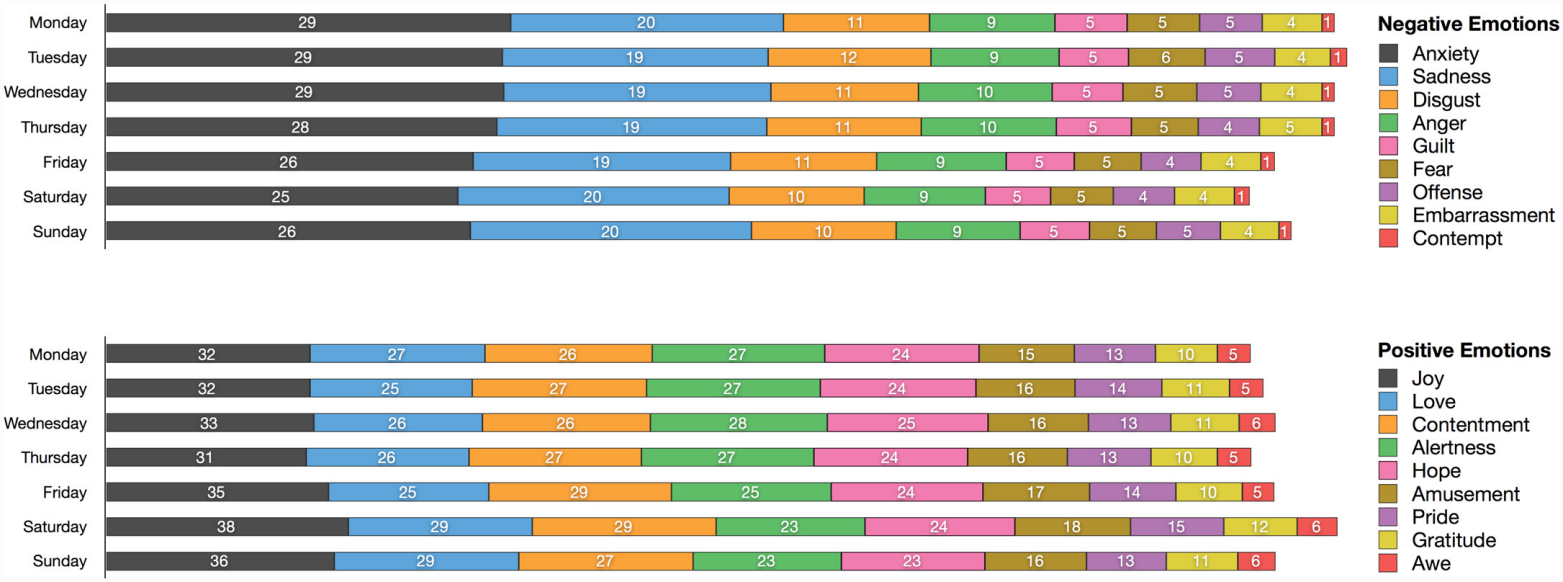
Experience of positive and negative emotions by day of the week.

In order to provide a detailed account of emotion in everyday life, we further broke down our results by reporting the frequency of emotions across the different days of the week and time of the day. Because relatively few people provided emotion reports from 11PM to 5AM (all these measurement times had fewer than 1,000 reports; see Fig 1), which might bias the frequency estimates, we report frequency data from 6AM to 10PM only. As depicted in Fig 2, people experience fewer negative emotions and more positive emotions on the weekend, and particularly Saturday. The relative frequency of specific emotions did not change much across the different days of the week.

**Fig 3:**
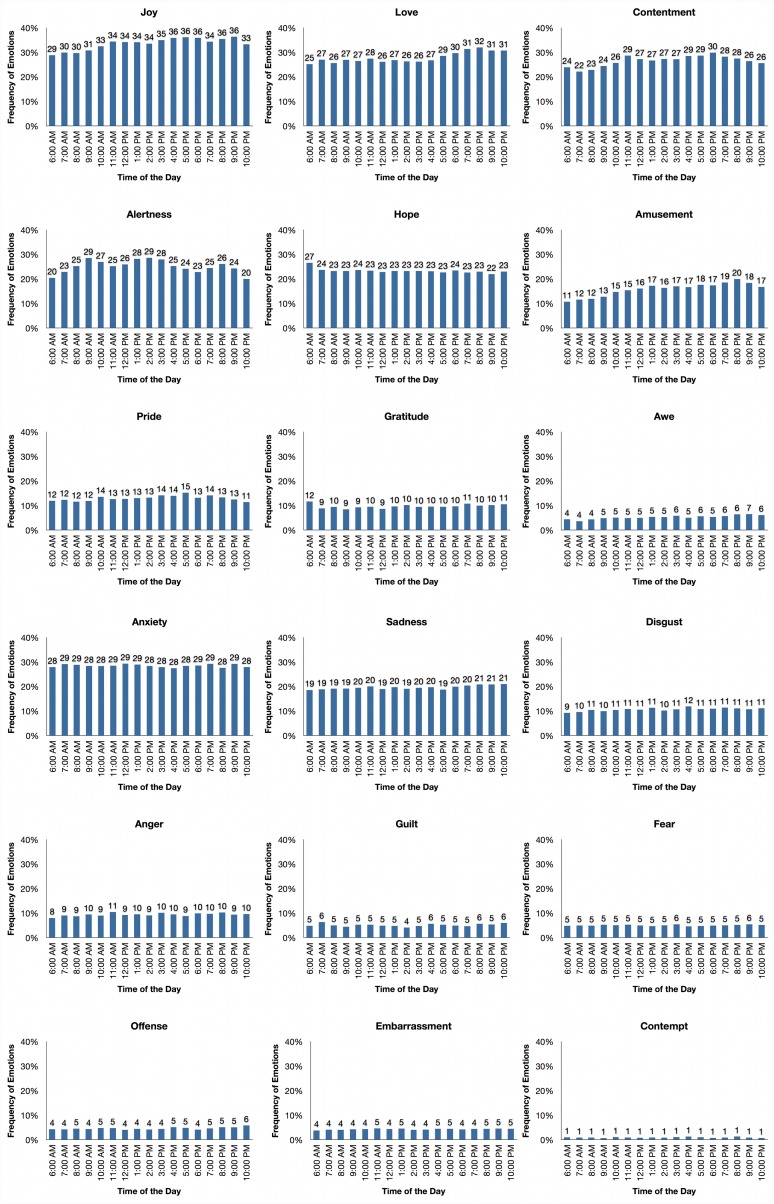
Emotional experience by time of day per emotion.

**Fig 4:**
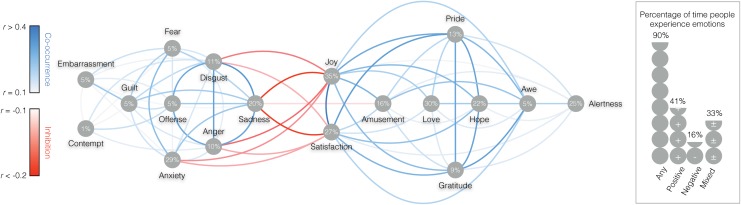
Frequency and centrality of emotions in everyday life. The line colors between specific emotions represent the extent to which emotions tend to co-occur (blue hues) or inhibit each other (red hues). The numbers in the grey dots underneath specific emotions represents their frequency of occurrence in the sample. The right panel represents the percentage of times respondents reported experiencing any, positive, negative, or mixed emotions.

Regarding the experience of specific positive emotions across different times of the day, Fig 3 shows that joy, love, amusement, and awe slightly increased throughout the day to peak around 8 or 9PM. Alertness seems to follow a more cyclic pattern with increases in frequency an hour or two after traditional meal times. Finally, contentment, pride, gratitude, and awe remain relatively stable throughout the day and do not seem to follow a clear temporal pattern. In contrast with positive emotions and as seen in Fig 4, we found very little fluctuation of the frequency of the specific negative emotions across different times of the day. In fact, the magnitude of temporal fluctuations for positive emotions—measured by the average standard deviation across hours of the day for the nine positive emotions—was over three times larger than the magnitude of temporal fluctuations for negative emotions (Mean SD_positive_ = .017 and Mean SD_negative_ = .006).

Gender differences: In order to explore potential gender differences in the frequency of emotional experience in everyday life, we analyzed the data separately for men and women. Table 3 displays the experience of emotions in everyday life for men and women separately. It seems that in general, men and women are very similar in terms of the frequency with which they report experiencing emotions in daily life (89.93% vs. 89.92%, respectively). However, men report experiencing only positive emotions more frequently (45.30%) than women (38.96%). In terms of specific positive emotions, men tend to report experiencing joy, satisfaction, alertness, amusement, pride, and awe more often than women. On the other hand, women more frequently report experiencing only negative emotions (16.83%), compared to men (14.02%). In terms of specific negative emotions, women tend to report experiencing more anxiety, sadness, disgust, anger, fear, offense, and contempt than men do. Thus, although the data suggest that women and men might be as likely to experience emotions in everyday life, women tend to report experiencing slightly more negative emotions than men do.

**Table 3:** Emotion frequency by gender.

	Women				Men			
Emotion	Frequency (% time)	95% CI Lower Bound	95% CI Upper Bound	Degree Centrality	Frequency (% time)	95% CI Lower Bound	95% CI Upper Bound	Degree Centrality
Joy	34.02	33.36	34.67	2.92	36.07	34.86	37.28	3.03
Love	30.10	29.30	30.90	1.33	29.56	28.22	30.91	1.43
Anxiety	30.31	29.59	31.04	1.68	26.13	24.95	27.31	1.84
Satisfaction	25.85	25.21	26.48	2.68	30.59	29.45	31.74	2.81
Alertness	23.06	22.31	23.81	1.29	27.44	26.36	28.52	1.35
Hope	22.34	21.70	22.99	2	22.48	21.38	23.58	2.22
Sadness	21.30	20.69	21.92	2.5	17.12	16.12	18.13	2.71
Amusement	15.78	15.15	16.41	1.78	18.10	17.20	19.00	1.79
Pride	12.23	11.66	12.81	2.16	15.73	14.77	16.69	2.36
Disgust	11.68	11.23	12.13	2.27	10.46	9.69	11.22	2.49
Anger	10.27	9.86	10.67	2.19	8.51	7.85	9.17	2.29
Gratitude	9.18	8.73	9.62	2.07	8.33	7.41	9.25	2.18
Guilt	5.43	5.12	5.74	1.53	5.57	5.00	6.13	1.81
Fear	5.68	5.34	6.02	1.53	4.49	3.97	5.01	1.73
Awe	5.09	4.70	5.48	1.87	5.31	4.62	6.00	1.96
Offense	5.19	4.90	5.49	1.75	4.13	3.69	4.58	1.8
Embarrassment	4.57	4.29	4.85	1.12	4.67	4.18	5.16	1.41
Contempt	0.97	0.81	1.14	0.85	1.35	1.08	1.61	1.45
Positive only	38.96	38.11	39.80	—	45.30	43.98	46.61	—
Negative only	16.83	16.30	17.35	—	14.02	13.15	14.89	—
Mixed emotion	33.81	33.10	34.51	—	30.51	29.32	31.70	—
ANY EMOTION	89.92	89.38	90.47	—	89.93	89.02	90.84	—

### Centrality of Emotions

#### Emotions in general

The aforementioned results tell us how ubiquitous, in terms of frequency, various specific emotions are in people’s daily lives. But emotions might also differ in how they relate to other distinct emotions within the emotional network. In other words, some emotions may typically stimulate or inhibit the experience of other emotions, whereas other emotions may typically be experienced in isolation, with no impact on the co-occurrence of other emotions. Human emotions can be represented as a network, wherein nodes represent specific emotions and the connections between them encode how likely emotions are to co-occur or inhibit one another. Graph theory can then be used to characterize and analyze this emotional network [31]. Specifically, we represented the network as a weighted undirected graph in which the strength of each connection was weighted by the correlation coefficient between the two emotions (seen as binary vectors equal to 1 when the emotion is experienced and to 0 otherwise). A strong positive edge in the network indicates two emotions that tend to co-occur, whereas a strong negative edge implies that the connected emotions inhibit one another.

We characterized the centrality of the different emotions in the network by their *Degree Centrality (DC)*, one of the most common measures of node centrality in a network [32]. The DC of an emotion is obtained by summing the absolute value of the weights of all the connections that this particular emotion makes with other emotions. Theoretically, the maximum value of the DC of an emotion in the network is 17, which would occur only if all emotions always co-occurred or perfectly inhibited one another. An emotion with a larger DC tends to co-occur with or inhibit other emotions in a more systematic pattern than an emotion with a lower DC. As depicted in Fig 4, our analysis revealed that emotions widely differed in how interconnected they were. The top 3 most central emotions was *joy*, followed by *satisfaction*, and *sadness*, while the least central emotion was *contempt*. The centrality of emotions in the network matters because it tells us which emotions are most likely to have an impact on one’s overall emotional life. In particular, a visual examination of the different interconnections reveals three broad categories of emotions. The first category consists of emotions that are strongly connected to several other emotions, including emotions of *opposite valence*. Following the standard terminology in network analysis [31], we refer to them as “connector emotions”. For instance, *joy* and *satisfaction* (and *amusement* to a lesser extent) are strongly connected to many other positive emotions such as *pride* and *gratitude*, but also to negative emotions such as *sadness*, *anxiety*, *disgust*, and *anger*. Note, however, that both *joy* and *satisfaction* tend to co-occur with many positive emotions, while they tend to inhibit the co-occurrence of negative emotions. Increasing *joy* and *satisfaction* may therefore be used as a buffer against these negative emotional states. Conversely, *sadness*, *anxiety*, *disgust*, and *anger* are also connector emotions, as they tend to co-occur with other negative emotions such as *guilt* and *fear*, while their experience tends to inhibit feelings of *joy* and *satisfaction*. As a second category we define “provincial emotions”, that is, emotions that are strongly connected to several other emotions, but only of the *same valence*. For instance, *love*, *gratitude*, *pride*, and *awe* are strongly connected to many other positive emotions but do not inhibit negative emotions. The same holds for *offense*, *fear*, and *guilt*, which are interconnected but do not inhibit positive emotions. Finally, we define “distal emotions”, that is, emotions that rarely co-occur with or inhibit other emotions. This is the case for *alertness*, *embarrassment*, and *contempt*, which seem to be experienced relatively independently from other emotions.

Finally, we note that the frequency of occurrence of specific emotions and their centrality in the emotional ecosystem are relatively independent. For example, *alertness* is frequently reported but largely disconnected from other emotions. Indeed, the data show that frequency and centrality of emotions were not strongly related, 95% CI (*r* = -.05, *r* = .75).

### Gender differences

**Fig 5:**
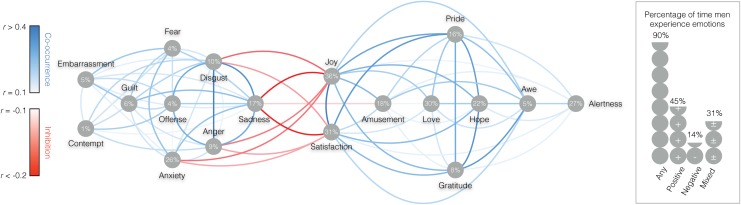
MEN: Frequency and centrality of emotions in everyday life. The line colors between specific emotions represent the extent to which emotions tend to co-occur (blue hues) or inhibit each other (red hues). The numbers in the grey dots underneath specific emotions represents their frequency of occurrence in the sample. The right panel represents the percentage of times respondents reported experiencing any, positive, negative, or mixed emotions.

**Fig 6:**
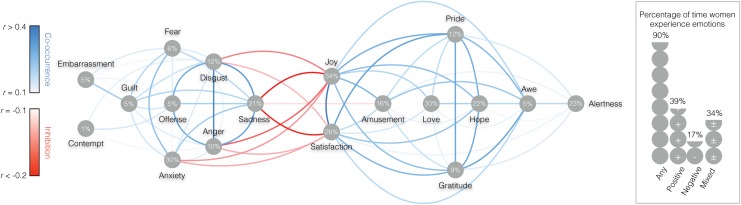
WOMEN: Frequency and centrality of emotions in everyday life. The line colors between specific emotions represent the extent to which emotions tend to co-occur (blue hues) or inhibit each other (red hues). The numbers in the grey dots underneath specific emotions represents their frequency of occurrence in the sample. The right panel represents the percentage of times respondents reported experiencing any, positive, negative, or mixed emotions.

In order to explore potential gender differences in the centrality of the different emotions in the network, we analyzed the data separately for men and women (see Table 3). Figs 5 and 6 display the interconnections of the 18 emotions for men and women, respectively. Emotions were, on average, significantly more strongly interconnected for men than for women, as indicated by a paired sample t-test on the centrality of emotions, *t*(17) = 5.60, *p* < .001, *d* = 1.39. These findings dovetail with previous research showing that men tend to be lower in emotional awareness [33] and have a less diverse emotional life [34].

## Discussion

We sought to capture and characterize people’s everyday emotional experiences through an experience sampling smartphone application. Our findings revealed that everyday human life is profoundly emotional: people reported experiencing at least one emotion 90% of the time. Positive emotions were reported over 2.5 times more frequently than negative emotions. This finding is consistent with previous studies that aimed to capture everyday emotional experience [13,14,16,35]. We also found that people indicated simultaneously experiencing both negative and positive emotions a substantial amount of the time, which extends laboratory studies on mixed emotions [36]. Finally, an examination of the interconnections within the emotional network provided the first evidence that distinct emotions can be characterized in three broad types depending on whether they interact with emotions of the same and opposite valence (connector emotions), of the same valence only (provincial emotions), or do not interact with other emotions (distal emotions). We believe these findings break new grounds in two important ways.

First, future research may draw from our frequency findings to determine which particular emotions deserve more research attention. Specifically, whereas some infrequently experienced emotions have received much research attention (e.g., fear is experienced 5% of the time but according to Google Scholar has been the focus of over 100,000 articles), some frequently experienced emotions might be relatively under-researched (e.g., sadness is experienced 20% of the time and has been the focus of less than 3,000 articles on Google Scholar).

Second, future research may use our centrality findings to determine which particular emotions could be used as leverage in psychological interventions. Specifically, our network analyses suggest that some positive emotions (e.g., joy and satisfaction) are likely to inhibit the occurrence of many negative emotions, whereas other positive emotions (e.g., hope and gratitude) do not appear to show these buffering properties. A growing number of interventions promote the cultivation of specific emotional states like hope [37] and gratitude [7]. Provided replication in other countries and cultures, our study suggests that designing interventions around the cultivation of buffer emotions might be an even more effective strategy. Furthermore, our novel network approach to emotions may set the stage for mapping out the emotions network for different pathologies. One can imagine, for example, that the connector emotions in the emotional network of people suffering from unipolar depression might substantially differ from the connector emotions in the emotional network of people suffering from bipolar depression or generalized anxiety. Identifying the key emotional states that are more likely to alleviate psychological suffering for different individuals or groups of individuals may provide critical insights into how increase their well-being.

### Limitations and future research

Although our findings break new grounds in several ways, the present research also suffers from several limitations, which should be addressed in future studies. First, although our smartphone application was designed to capture the widest possible range of episodes of daily life, participants had the opportunity to skip questionnaires. It is therefore theoretically possible that several emotions might be under- or over-represented, as different specific emotions might have different effect on people’s motivation to respond to the questionnaire prompts. In addition, responses to the app prompts were not evenly distributed throughout the day: essentially, the workday was oversampled. Also, it is theoretically possible that the specific emotion respondents experienced affected their likelihood of responding to the prompt. A second limitation lies in the dichotomous format of our emotion items. We chose to present our 18 emotions as a non-exclusive choice list. This allowed us to collect a very large amount of data, since participants would have been unlikely to respond as often if they had to rate each of the 18 emotions on continuous scales several times a day. However, we cannot exclude the possibility that dichotomous items required respondents to make idiosyncratic judgments about when to report an emotion as being present, which might have increased demand characteristics and the frequency of emotion reporting. Finally, although our choice of emotions respondents were presented with was based on careful consideration of the literature, the selected emotions may still be argued to differ in the extent to which they contain emotional vs. cognitive and attributional components. Future research may investigate to what extent emotion frequency and centrality are related to the relative importance of emotional vs. cognitive and attributional components of specific emotions.

Although our aim in the current research was not to focus on the structure of affect, our data indeed suggest that people generally experience pleasant emotions in the presence of pleasant emotions and unpleasant emotions in the presence of unpleasant emotions. In that sense, our findings align with one of the most well-known models on the structure of affect—the circumplex model [18–21]. However, per the circumplex model, one would expect that all (or at least most) positive emotions would correlate negatively with negative emotions, and vice versa. In our data, a large number of emotions do not correlate with opposite-valence emotions (e.g., fear and gratitude). If positive and negative emotions were on the same valence dimension, we would observe negative correlations between all negative emotions and all positive emotions. For an experience sampling study on everyday emotions to be able to really address the circumplex model, measures of both valence and arousal should be included. Also, our finding that mixed emotions occur 33% of the time is inconsistent with the circumplex model’s assumption that positive and negative affect are bipolar. Future research may take a more systematic approach to this issue and include valence as well as arousal measures.

Regarding our finding that some emotions inhibit each other, we feel we should clarify what ‘inhibition’ means in our cross-sectional dataset. When two emotions are negatively correlated, this either means that (1) one emotion inhibits the other, or (2) another variable inhibits one and stimulates the other emotion. In both cases, inhibition occurs, but information about the direction is lacking.

Further work is also needed to explore the connections between our findings about the centrality of emotions—and in particular the fact that men’s emotions were more strongly interconnected than women’s emotions—and related concepts of emotion granularity and emodiversity. Emotion granularity refers to people’s tendency to *represent* emotional experiences with precision and specificity (rather than as global states) [38,39]. Emodiversity refers to people’s tendency to *experience* diverse emotions in terms of variety and relative abundance [35]. Future research could try to disentangle whether our findings can be explained because men have less emotion granularity (i.e., men select various specific emotions at the same time simply because they feel globally good or bad) or because men have more emodiversity (i.e., men select various specific emotions at the same time simply because they do feel a subtle mix of different emotions).

Although research on emotions is abundant, knowledge about emotions in everyday life has been particularly scarce. Providing both basic foundations and novel tools, these findings provide evidence that emotions are ubiquitous in everyday life and can exist both in concert and distinctly, which has important implications for emotional interventions and theory.

## Supporting Information

S1 Table Co-occurrence of emotions. (DOCX)

## References

[1] Berkowitz L. On the formation and regulation of anger and aggression: A cognitive-neoassociationistic analysis. Am Psychol.1990; 45: 494–503. 2186678 10.1037//0003-066x.45.4.494

[2] Frijda NH. The emotions. Cambridge: Cambridge University Press; 1986.

[3] Smith CA, Lazarus RS. Appraisal components, core relational themes, and the emotions. Cogn Emot. 1993;7: 295–323.

[4] Markus HR, Kitayama S. Culture and the self: Implications for cognition, emotion, and motivation. Psychol Rev. 1991;98: 224–253.

[5] Chaiken S, Eagly AH. Heuristic and systematic information processing within and beyond the persuasion context In: Uleman J, Bargh JA, editors. Unintended thought. New York: Guilford;1989 pp.212–252.

[6] Forgas JP. Mood and judgment: The affect infusion model (AIM). Psychol Bull. 1995;117: 39–66. 7870863 10.1037/0033-2909.117.1.39

[7] Wood AM, Froh JJ, Geraghty AW. Gratitude and well-being: A review and theoretical integration. Clin. Psychol Rev. 2010;30: 890–905. 10.1016/j.cpr.2010.03.005 20451313

[8] Rudd M, Vohs KD, Aaker J. Awe expands people’s perception of time, alters decision making, and enhances well-being. Psychol Sci. 2012;23: 1130–1136. 10.1177/0956797612438731 22886132

[9] Fredrickson BL. Love 2.0: Creating Happiness and Health in Moments of Connection. New York: Penguin; 2013.

[10] Emmons RA, McCullough ME. Counting blessings versus burdens: an experimental investigation of gratitude and subjective well-being in daily life. J Pers Soc Psychol. 2003;84, 377–389. 12585811 10.1037//0022-3514.84.2.377

[11] Hutcherson CA, Seppala EM, Gross JJ. Loving-kindness meditation increases social connectedness. Emotion. 2008;8: 720–724. 10.1037/a0013237 18837623

[12] Lerner JS, Li Y, Valdesolo P, Kassam K. Emotion and decision making. Annu Rev Psychol. 2015;66: in press. 10.1146/annurev-psych-010213-115043 25251484

[13] Carstensen LL, Pasupathi M, Mayr U, Nesselroade JR. Emotional experience in everyday life throughout the adult life span. J Pers Soc Psychol. 2000; 799: 644–655. 11045744

[14] Heiy JE, Cheavens JS. Back to basics: A naturalistic assessment of the experience and regulation of emotion. Emotion, 2014;14: 878–891. 10.1037/a0037231 24999913

[15] Vansteelandt K, Van Mechelen I, Nezlek JB. The co-occurrence of emotions in daily life: A multilevel approach. J Res Pers. 2005;39: 325–335.

[16] Zelenski JM, Larsen RJ. The distribution of basic emotions in everyday life: A state and trait perspective from experience sampling data. J of Res Pers. 2000;34: 178–197.

[17] aan het Rot M, Hogenelst K, Schoevers RA. Mood disorders in everyday life: A systematic review of experience sampling and ecological momentary assessment studies. Clin Psychol Review. 2012; 32: 510–523. 10.1016/j.cpr.2012.05.007 22721999

[18] Schlosberg H. The description of facial expressions in terms of two dimensions. J Exp Psychol. 1952;44: 229–237. 13000062 10.1037/h0055778

[19] Russell JA. A circumplex model of affect. J Pers Soc Psychol. 1980;39: 1161–1178.

[20] Remington NA, Fabrigar LR, Visser PS. Reexamining the circumplex model of affect. J Pers Soc Psychol. 2000;79: 286–300. 10948981 10.1037//0022-3514.79.2.286

[21] Russell JA, Barrett LF. Core affect, prototypical emotional episodes, and other things called “emotion”: Dissecting the elephant. J Pers Soc Psychol.1999;76: 805–819. 10353204 10.1037//0022-3514.76.5.805

[22] Tichy NM, Tushman ML, Fombrun C. Social network analysis for organizations. Acad Manage Review. 1979; 4: 507–519.

[23] Izard CE. Human emotions. New York: Plenum Press; 1977.

[24] Philippot P, Schaefer A, Herbette G. Consequences of specific processing of emotional information: Impact of general versus specific autobiographical memory priming on emotion elicitation. Emotion. 2003;3:270–283. 14498796 10.1037/1528-3542.3.3.270

[25] Barrett LF. Are emotions natural kinds? Perspect Psychol Sci. 2006; 1: 28–58. 10.1111/j.1745-6916.2006.00003.x 26151184

[26] Philippot P. Inducing and assessing differentiated emotion-feeling states in the laboratory. Cognition Emotion. 1993; 7: 171–193. 27102736 10.1080/02699939308409183

[27] Youngstrom EA, Green KW. Reliability generalization of self-report of emotions when using the Differential Emotions Scale. Educ Psychol Meas. 2003; 63: 279–295.

[28] Fredrickson BL, Tugade MM, Waugh CE, Larkin GR. What good are positive emotions in crises? A prospective study of resilience and emotions following the terrorist attacks on the United States on September 11th, 2001. J Pers Soc Psychol. 2003; 84: 365–376. 12585810 10.1037//0022-3514.84.2.365 PMC2755263

[29] Schaefer A, Nils F, Sanchez X, Philippot P. Assessing the effectiveness of a large database of emotion-eliciting films: A new tool for emotion researchers. Cognition Emotion. 2010; 24: 1153–1172.

[30] Bigot R, Croutte P, Daudey E. La diffusion des technologies de l’information et de la communication dans la société française. Paris: Conseil Général de l’Economie, de l’Industrie, de l’Energie et des Technologies (CGEIET); 2013.

[31] Strogatz SH. Exploring complex networks. Nature. 2001;410: 268–276. 11258382 10.1038/35065725

[32] Van den Heuvel MP, Sporns O. An anatomical substrate for integration among functional networks in human cortex. J Neurosci. 2013;33: 14489–14500. 10.1523/JNEUROSCI.2128-13.2013 24005300 PMC6618386

[33] Barrett LF, Lane R, Sechrest L, Schwartz G. Sex differences in emotional awareness. Pers Soc Psychol B. 2000;26: 1027–1035.

[34] Quoidbach J, Gruber J, Mikolajczak M, Kogan A, Kotsou I, Norton MI. Emodiversity and the emotional ecosystem. J Exp Psychol Gen. 2014; 143: 2057–2066. 10.1037/a0038025 25285428

[35] Diener E, Kanazawa S, Suh EM, Oishi S. Why people are in a generally good mood. Pers Soc Psychol Review. 2014; 1–22. 10.1177/1088868314544467 25253069

[36] Larsen JT, McGraw AP. Further evidence for mixed emotions. J Pers Soc Psychol. 2011; 100: 1095–1110. 10.1037/a0021846 21219075

[37] Weis R, Speridakos EC. A meta-analysis of hope enhancement strategies in clinical and community settings. Psychol Well Being. 2011;1: 1–16 22328976

[38] Barrett LF, Gross J, Christensen TC, Benvenuto M. Knowing what you're feeling and knowing what to do about it: Mapping the relation between emotion differentiation and emotion regulation. Cognition Emotion. 2001;15: 713–724.

[39] Tugade MM, Fredrickson BL, Barrett LF. Psychological resilience and positive emotional granularity: Examining the benefits of positive emotions on coping and health. J Pers. 2004;72: 1161–1190. 15509280 10.1111/j.1467-6494.2004.00294.x PMC1201429

