# Supplementary material for: Emotions in Everyday Life
Source: PLoS One. 2015 Dec 23;10(12):e0145450. doi: 10.1371/journal.pone.0145450 (PMC4689475; doi:10.1371/journal.pone.0145450)
Supplement: S1 Table — (DOCX) [file pone.0145450.s001.docx]

**S1 Table 1: Co-occurrence of emotions. The grey box highlights correlations between mixed emotions; mixed emotions co-occurrences of *r* ≥ .05 are in bold; **p* < .05; ***p* < .01.**

|  | 1 | 2 | 3 | 4 | 5 | 6 | 7 | 8 | 9 | 10 | 11 | 12 | 13 | 14 | 15 | 16 | 17 | 18 |
| --- | --- | --- | --- | --- | --- | --- | --- | --- | --- | --- | --- | --- | --- | --- | --- | --- | --- | --- |
| 1. Anxiety | ⎯ |  |  |  |  |  |  |  |  |  |  |  |  |  |  |  |  |  |
| 2. Contempt | -.04** | ⎯ |  |  |  |  |  |  |  |  |  |  |  |  |  |  |  |  |
| 3. Offense | -.10** | -.13** | ⎯ |  |  |  |  |  |  |  |  |  |  |  |  |  |  |  |
| 4. Guilt | -.06** | -.06** | -.09** | ⎯ |  |  |  |  |  |  |  |  |  |  |  |  |  |  |
| 5. Disgust | -.18** | -.10** | -.20** | -.07** | ⎯ |  |  |  |  |  |  |  |  |  |  |  |  |  |
| 6. Fear | -.07** | -.06** | -.08** | -.11** | -.05** | ⎯ |  |  |  |  |  |  |  |  |  |  |  |  |
| 7. Embarrassment | -.01** | -.05** | -.07** | -.15** | -.03** | -.09** | ⎯ |  |  |  |  |  |  |  |  |  |  |  |
| 8. Sadness | -.22** | -.02* | -.10** | -.08** | -.16** | -.08** | -.01 | ⎯ |  |  |  |  |  |  |  |  |  |  |
| 9. Anger | -.14** | -.08** | -.20** | -.05** | -.26** | -.04** | -.00 | -.15** | ⎯ |  |  |  |  |  |  |  |  |  |
| 10. Pride | -.01 | -.02** | -.03** | -.00 | -.01 | -.01 | -.02** | -.06** | -.02** | ⎯ |  |  |  |  |  |  |  |  |
| 11. Love | -**.06**** | -.00 | -.00 | -**.07**** | -.03** | -**.08**** | -.01 | -**.09**** | -**.05**** | -.07** | ⎯ |  |  |  |  |  |  |  |
| 12. Hope | -**.05**** | -.02** | -.00 | -.02* | -.04** | -**.06**** | -.02* | -.05** | -.03** | -.18** | -.03** | ⎯ |  |  |  |  |  |  |
| 13. Gratitude | -.02** | -.01 | -.02** | -**.06**** | -.02* | -.02* | -**.06**** | -.02** | -.01 | -.20** | -.03** | -.15** | ⎯ |  |  |  |  |  |
| 14. Joy | -.00 | -.01 | -.04** | -.02* | -.09** | -.05** | -.03** | -.16** | -.11** | -.20** | -.02* | -.11** | -.15** | ⎯ |  |  |  |  |
| 15. Satisfaction | -.01 | -.00 | -.02* | -.00 | -.07** | -.05** | -.02** | -.13** | -.08** | -.24** | -.02* | -.10** | -.17** | -.32** | ⎯ |  |  |  |
| 16. Awe | -.01 | -.01 | -.01 | -.03** | -.00 | -.03** | -**.05**** | -.00 | -.00 | -.17** | -.06** | -.11** | -.22** | -.14** | -.13** | ⎯ |  |  |
| 17. Amusement | -.02** | -.01 | -.00 | -.00 | -.01 | -.00 | -**.07**** | -.07** | -.05** | -.10** | -.05** | -.00 | -.07** | -.19** | -.10** | -.15** | ⎯ |  |
| 18. Alertness | -.00 | -.01 | -.01 | -.01 | -.01 | -.01 | -.02** | -.02* | -.02** | -.03** | -.18** | -.05** | -.03** | -.04** | -.00 | -.12** | -.01 | ⎯ |
|  |  |  |  |  |  |  |  |  |  |  |  |  |  |  |  |  |  |  |
